# The application of transanal total mesorectal excision for patients with middle and low rectal cancer: A systematic review and meta-analysis

**DOI:** 10.1097/MD.0000000000011410

**Published:** 2018-07-13

**Authors:** Dongping Hu, Penghui Jin, Lidong Hu, Wenhan Liu, Weisheng Zhang, Tiankang Guo, Xiongfei Yang

**Affiliations:** a Gansu Provincial Hospital; b Clinical Medical College, Gansu University of Chinese Medicine, Lanzhou, Gansu, China.

**Keywords:** laparoscopic, meta-analysis, rectal cancer, total mesorectal excision, transanal

## Abstract

**Background:**

Recently, in order to overcome the shortcomings of laparoscopic surgery in the treatment of low rectal cancer, a new kind of surgical procedure, transanal total mesorectal excision (TaTME), has rapidly become a research hotspot in the field of rectal cancer surgery study. Our study aimed to evaluate the efficacy and safety of transanal total mesorectal excision (TaTME) for the patients with rectal cancer.

**Methods:**

Relevant studies were searched from the databases of the Cochrane Library, PubMed, Embase, Web of science. All relevant studies were collected to evaluate the efficacy and safety of TaTME for patients with rectal cancer. The quality of the included studies was assessed by the Newcastle-Ottawa Quality Assessment Scale (NOS) and Cochrane Library Handbook 5.1.0. Data analysis was conducted using the Review Manager 5.3 software.

**Results:**

Thirteen studies including 859 patients were included in our analysis. In terms of efficacy, compared with laparoscopic total mesorectal excision (LaTME), meta-analysis showed that the rate of complete tumor resection increased and the risk of positive circumferential margins decreased in the TaTME group. For complete tumor resection and positive circumferential margins in the TaTME group, the odds ratios (ORs) and 95% confidence intervals (CIs) were 1.93 and 1.09 to 3.42 (*P* = .02) and 0.43 and 0.22 to 0.82 (*P* = .01), respectively. Concerning safety, results showed that the rates of postoperative complications were similar in the 2 groups, and differences in the risk of ileus and anastomotic leakage were not statistically significant (OR = 0.75, 95%CI = 0.51–1.09, *P* = .13; OR = 0.91, 95%CI = 0.46–1.78, *P* = .78; OR = 0.79, 95%CI = 0.45–1.38, *P* = .40).

**Conclusions:**

The results of this meta-analysis show that TaTME is associated with a reduced positive circumferential resection margin (CRM) rate, and could achieve complete tumor resection and improved the long-term survival in patients with mid- and low-rectal cancer.

## 1 Introduction

Rectal cancer is one of the most common malignant tumors, with a trend towards occurrence in younger patients.^[1,2]^ The morbidity rate is also increasing every year; this is particularly true among patients with low- and mid-rectal cancer, accounting for 70% to 80% of all rectal cancer cases. In recent years, comprehensive treatment has continued to be based on surgical resection. Since Heald et al^[3]^ proposed the concept of total meso-rectal excision (TME) in 1982, a large number of studies have shown that TME could effectively reduce the recurrence of rectal cancer after surgery and significantly improve patients’ quality of life; it has thus become a standard procedure for the treatment of rectal cancer.

With further developments in medical technology and minimally invasive surgery, open surgery has been gradually replaced by laparoscopic techniques. Several randomized controlled trials (RCTs)^[4–7]^ demonstrated that laparoscopic total mesorectal excision (LaTME) has a significant advantage in both short-term and long-term outcomes, as compared with traditional open TME. However, LaTME has some limitations in mid- and low-rectal cancer operations, such as TME quality control, high risk of positive circumferential resection margin (CRM) and distal resection margin (DRM), and greater difficulties in patients with a contracted pelvis, prostatic hypertrophy, and obesity.^[8]^ Recently, in order to overcome the shortcomings of laparoscopic surgery in the treatment of low rectal cancer, a new kind of surgical procedure, transanal total mesorectal excision (TaTME), has rapidly become a research hotspot in the field of rectal cancer surgery study.^[4,5]^

Since 2010 Sylla et al^[9]^ first reported that laparoscopy can be applied for TaTME in the resection of rectal cancer, a few reports have demonstrated TaTME to have a good efficacy for the treatment of rectal cancer over the short and medium term. However, it still remains controversial whether TaTME is superior to LaTME in terms of oncologic and perioperative outcomes. Therefore, based on the evidence-based approach, this dissertation attempts to compare TaTME and LaTAM in terms of the oncologic and perioperative outcomes of patients with mid- and low-rectal cancer in order to provide clinical reference.

## 2 Materials and methods

### 2.1 Search strategy

In this paper, multiple foreign language databases including PubMed, Embase, and Web of Science were retrieved systematically for all articles with title or abstract from inception to Feb 15, 2017. And The Cochrane Library was retrieved to No. 1 in 2017. The MeSH and main keywords were as follows “rectal neoplasm OR rectal tumor OR rectal cancer OR cancer of rectum OR rectum neoplasm OR rectum cancer OR rectum tumor,” “transanal OR transanal minimally invasive surgery OR TAMIS OR transanal endoscopic microsurgery OR TEM OR transanal specimen extraction OR natural orifice specimen extraction OR NOSE OR natural orifice transluminal endoscopic surgery OR NOTES OR Transanal Endoscopic Surgical Procedure OR peritoneal,” “total mesorectal excision OR TME OR proctectomy OR proctocolectomy.” Based on these MeSH and main keywords, we formulated the search strategy (for PubMed) as shown in Box 1.

**Box 1.**
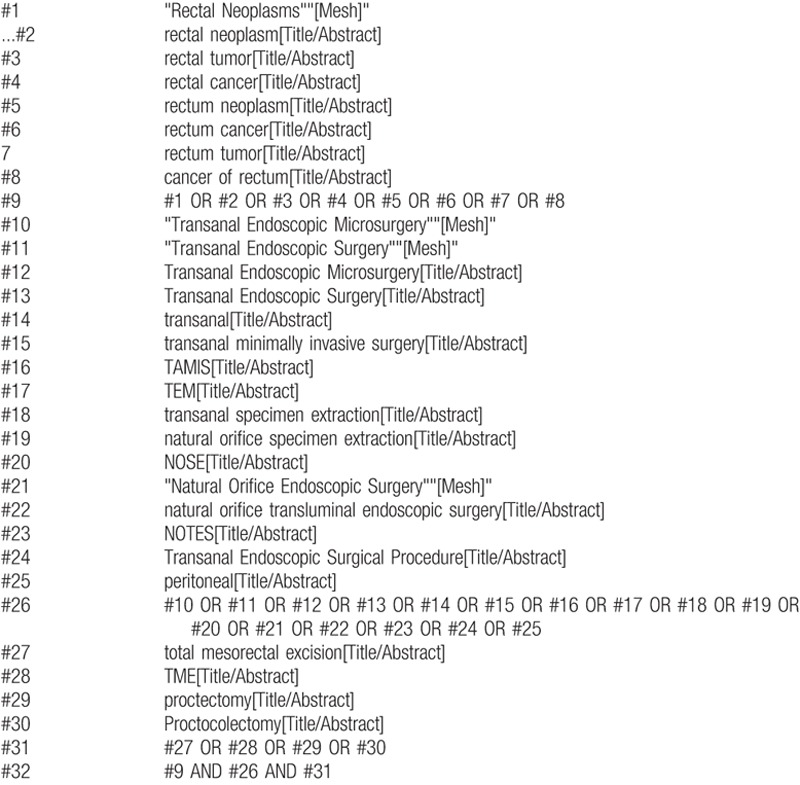
PubMed search strategy

### 2.2 Inclusion and exclusion criteria

Inclusion criteria of this study were as follows: enrollment of patients who were diagnosed with rectal cancer based on pathological examination; comparison of TaTME with LaTME for rectal cancer; reporting of the major outcome indicators. Exclusion criteria were as follows: reviews, meta-analysis, letters, case reports or conference abstracts; duplicate or repeat studies; studies on transanal extraction of other large bowel segments; non-human research.

### 2.3 Data extraction and quality assessment

According to the inclusion and exclusion criteria, 2 reviewers (PHJ and DPH) independently reviewed and assessed the risk bias of each included study. In addition, data extraction was performed independently, and the following information was collected: study characteristics: first author, publication date, country, study design, and number of patients enrolled in each group; age, sex, body mass index, tumor site (mid or low), American Society of Anesthesiologists (ASA) score, and neoadjuvant treatment; major outcome indicators: oncological outcomes (harvested lymph nodes, CRM, positive CRM, DRM, positive DRM, and perioperative outcomes [conversion, operation time, blood loss, ileus, mobilization of the splenic flexure, hospital stay, intraoperative complications, postoperative complications, and macroscopic quality of the mesorectum]). The quality of the matched case control (MCC) studies was evaluated by using the Newcastle-Ottawa Scale (NOS) criterion. The quality of RCTs was evaluated using Cochrane Library Handbook 5.1.0. All disagreements were resolved through discussion between the 2 reviewers (PHJ and DPH).

### 2.4 Statistical analysis

Continuous variables were analyzed by weighted mean difference (WMD) with a 95% confidence interval (CI). However, some studies did not report the mean and standard deviation (SD); in this cases, we used the median and range to calculate the mean and WMD. We used odds ratios (OR) and 95% CI to evaluate dichotomous variables. In addition, we used the *I*^2^ statistic to assess heterogeneity among studies. If *I*^2^ > 50%, significant heterogeneity among studies is present, and the random-effect model was used. Otherwise, the fixed effects model was used. All statistical values were computed with 95% CIs and the *P* value threshold for statistical significance was set at .05. All statistical analyses were performed using the Review Manager 5.3 software (Nordic Cochrane Centre, The Cochrane Collaboration, Copenhagen) and publication bias was tested using funnel plots.

## 3 Results

### 3.1 Study characteristics

We searched 1685 references initially by computer retrieval. A total of 347 duplicate articles were excluded by computer screening and 1299 unrelated articles were excluded after reading their titles and abstracts. Next, 26 studies were excluded from 39 potentially eligible studies by reading the full articles. Finally, 13 studies were included, which enrolled 859 patients (TaTME group = 414; LaTME groups = 445).^[10–22]^ There were 3 RCTs and 10 MCCs comparing TaTME with LaTME for rectal cancer. The baseline characteristics of the included studies are summarized in Table 1.

**Table 1:**
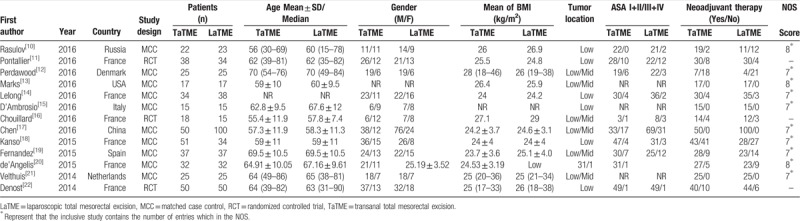
The baseline characteristics of the included studies.

**Figure 1:**
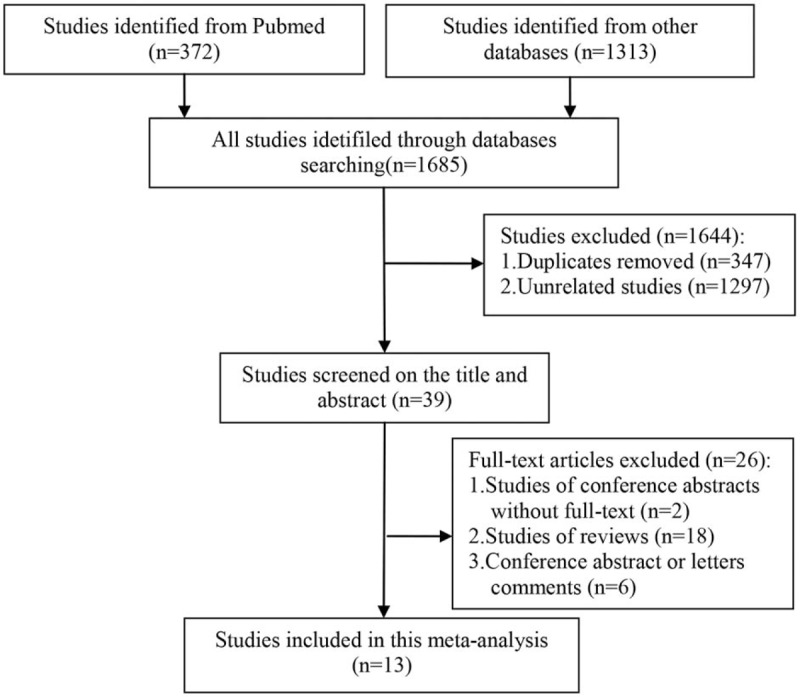
Screening flow chart for the included studies.

**Figure 2:**
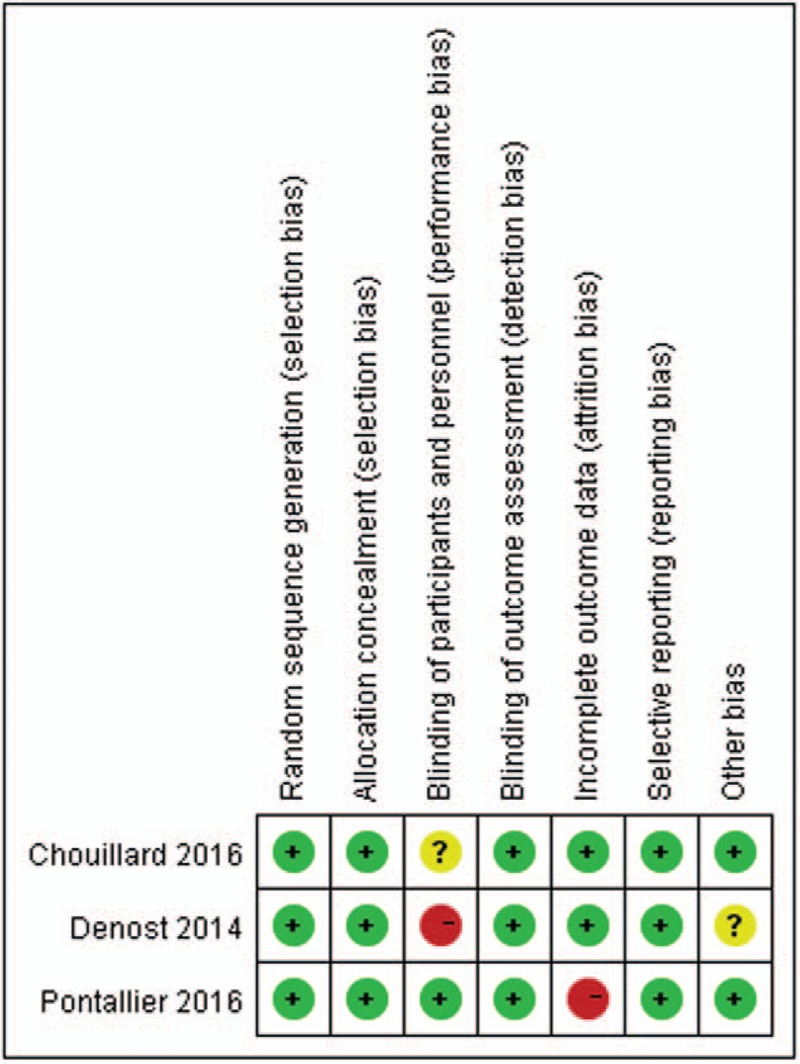
Quality assessment of 3 RCTs. RCTs = randomized controlled trials.

The quality assessment of all the MCCs were evaluated using NOS and the results ranged from 7 to 8 stars, this showed that the quality of the methodology was generally good (Table 1). The screening flow chart for the included studies is illustrated in Fig. 1. The quality assessment of the 3 RCTs is shown in Fig. 2. The overall quality of the 3 RCTs was better, but the sample size was small and a large number of multi-center, large sample size studies are needed.

### 3.2 Meta-analysis results

#### 3.2.1 Oncological meta-analysis results

**Figure 3:**
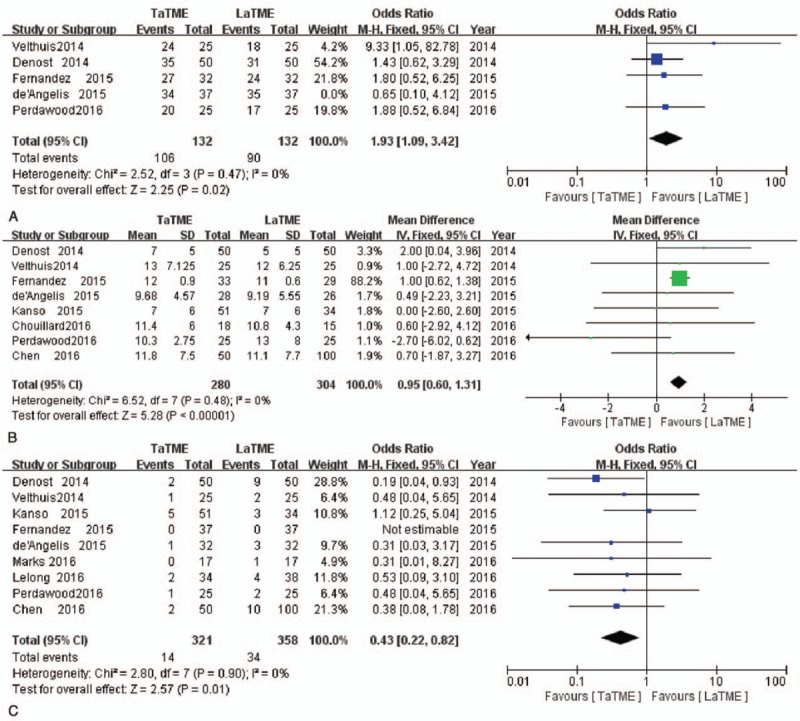
Forest plots of macroscopic quality of mesorectum (A); circumferential resection margin (B); positive circumferential resection margin (C).

All oncological meta-analysis results are shown in Table 2. Compared with the LaTME group, the results showed that macroscopic quality of the mesorectum was better than in the TaTME group, and this difference was statistically significant (OR = 1.93, 95%CI = 1.09–3.42, *P* = .02). A longer circumferential resection margin and a lower positive circumferential resection margin were identified in the TaTME group (WMD = 0.95, 95%CI = 0.60–1.31, *P* = .001 and OR = 0.43, 95%CI = 0.22–0.82, *P* = .01, respectively) (Fig. 3).

**Table 2:**
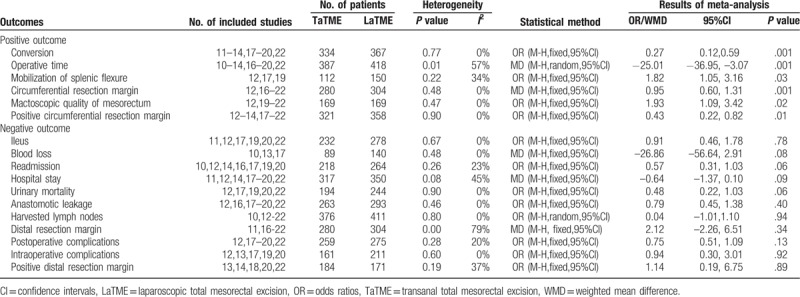
Meta-analysis results of TaTME compared with LaTME for rectal cancer.

In the TaTME group, a longer distal resection margin and a lower positive distal resection margin were identified, but the differences were not statistically significant (WMD = 2.12, 95%CI = –2.26–6.51, *P* = 0.34, *I*^2^ = 79%; OR = 1.14, 95%CI = 0.19–6.75, *P* = .89). However, with high heterogeneity of the distal resection margin, the result did not change by removing each study one at a time. The number of harvested lymph nodes was similar between the 2 groups (OR = 0.85, 95%CI = –2.97–4.66, *P* = .66, *I*^2^ = 94%). We found that when the study by D’Ambrosio was removed, heterogeneity was low (OR = 0.04, 95%CI = –1.01–1.10, *P* = 0.94, *I*^2^ = 0%).

#### 3.2.2 Perioperative meta-analysis results

**Figure 4:**
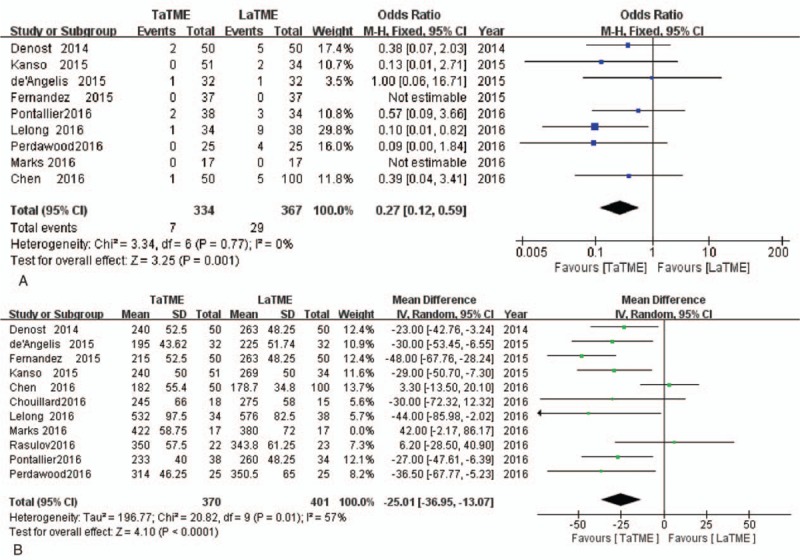
Forest plots of conversion (A); operative time (B).

All perioperative meta-analysis results are reported in Table 2. In terms of operative outcomes, the risk of intraoperative complications was similar between the 2 groups (OR = 0.85, 95%CI = −2.97–4.66, *P* = .66). Compared with the LaTME group, a lower rate of conversion and reduced operative time was identified in the TaTME group (OR = 0.27, 95%CI 0.12–0.59, *P* = .001 and WMD = –25.01, 95%CI –36.95, –3.07, *P* = .001, respectively) (Fig. 4). The TaTME group showed less blood loss than the LaTME group, but this difference was not statistically significant (WMD = –26.86, 95%CI = –56.64–2.91, *P* = .08).

In terms of postoperative outcomes, there were trends towards lower rates of postoperative complications, ileus, anastomotic leakage, reoperation, readmission, and postoperative hospital stay in the TaTME group than in the LaTME group, but all differences were not statistically significant.

### 3.3 Publication bias

**Figure 5:**
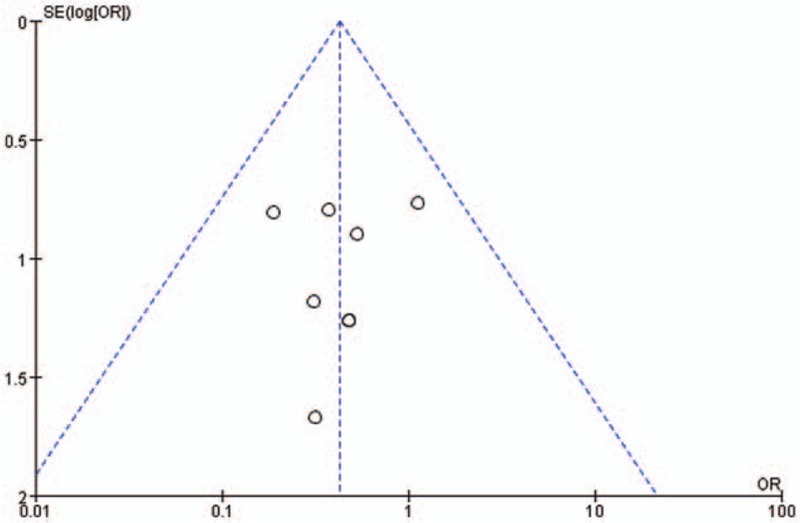
Funnel plot based on TaTME. TaTME = transanal total mesorectal excision.

A funnel plot based on the TaTME was generated to assess publication bias (Fig. 5). We detected no significant publication bias by visual inspection of the funnel plot.

## 4 Discussion

In order to prove whether TaTME is a better technique than LaTME, we evaluated the efficacy and safety of 2 surgical approaches for the treatment of rectal cancer based on an evidence-based medical meta-analysis. In terms of oncological outcomes, the results showed that the mesenchymal complete resection rate in the TaTME group was 1.93 times higher than that in the LaTME group; a better CRM and a lower rate of positive CRM were identified in the TaTME group than in the LaTME group. In terms of perioperative outcomes, the conversion rate and operation time in the TaTME group were significantly lower than those in the LaTME group, but the incidence of postoperative complications was similar between the 2 groups.

Some studies^[23,24]^ have demonstrated that CRM and positive CRM are important predictors of postoperative outcomes in patients with rectal cancer and represent strong predictors of whether the patients should receive postoperative adjuvant radiotherapy and chemotherapy. With the promotion and increased clinical application of TME and CRM, the overall survival of patients of rectal cancer is now similar to that of colon cancer. With to the aim of lowering the local recurrence rate and improving prognosis for patients with rectal cancer, the concept of CRM has been emphasized especially in patients with low rectal cancer, based on a standardized diagnosis and treatment process. Recently, CRM has become an important indicator that surgeons and pathologists take into consideration to determine the degree of radical surgery, representing a model for multidisciplinary collaboration to solve a scientific problem.

Our results confirmed that TaTME in the treatment of mid- and low-rectal cancer achieved a better CRM and lower positive CRM rate, which indirectly shows that TaTME could be used to achieve R0 resection. However, the results also showed that the effect of DRM and positive DRM were similar in the 2 groups and the difference was not statistically significant. In addition, there was large heterogeneity (*I*^2^ = 94%) between the included studies for the DRM, which might be due to differences in tumor location. Among the DRM studies, 3 studies^[18,20,22]^ included patients with only low rectal cancer and 5 studies^[12,16,17,19,21]^ included patients with mid- and low-rectal cancer. From this point of view, the differences in composition are significant between these 2 different locations, indirectly demonstrating that the location of the tumor may be a source of heterogeneity. Sylla et al^[9]^ also showed that the difference in distance from the tumor to the dentate line was statistically significant. Therefore, the real effect of TaTME in the outcome of DRM needs further verification with high quality RCTs.

Concerning perioperative outcomes, our analysis showed similar results in terms of readmission and hospital stay in 2 groups, but the results of conversion rate and operative time were lower in the TaTME group than in the. LaTME. Similarly, 1 study^[25]^ reported that there were no differences in the length of hospital stay and conversion rate in the TaTME and LaTME groups (*P* = .11). Pontallier et al^[11]^ and Sylla et al^[9]^ demonstrated that the surgical teams; TaTME coordination, and surgical skills may be important factors resulting in shorter surgical procedures. The main reason that TaTME had a lower conversion rate than LaTME was that TaTME could overcome adverse operative condition related to poor surgical fields due to pelvic stenosis, prostatic hypertrophy, mesorectal hypertrophy, or obesity. Therefore, TaTME can be used as a potentially reliable and effective alternative surgical method for patients with the above mentioned adverse factors and a high risk of rectal cancer.

TaTME surgery is an innovative surgical technique that conforms to the concept of natural orifice transluminal endoscopic surgery (NOTES), which is a “down-to-up” procedure completed through the anus and rectum natural orifice using vascular detachment, lymph node dissection, specimen resection, and digestive tract reconstruction. The safety of TaTME is also one of the focal points of our analysis. Our results showed that the same incidence of intraoperative and postoperative complications in the 2 groups, differences were not statistically significant. Results also indirectly suggested that the readmission rate between the 2 groups was also comparable.

In the subgroup analysis of postoperative complications, the results showed that the risk of ileus, anastomotic leakage and pelvic organ damage was similar between the 2 groups. One study^[25]^ showed that the incidence rate of urinary tract complications was lower in the TaTME group than in the LaTME group, but this difference was not statistically significant. Another study^[20]^ reported that there was no significant difference in gastrointestinal function between groups, but the TaTME group had the advantage of protection of sexual function. There was less data available concerning the long-term quality of life for patients treated with TaTME, 1 study^[26]^ reported short-term quality of life for 6 months after TaTME in patients with rectal cancer. Therefore, the real effect of TaTME in safety needs to be further verified with the high quality RCTs.

There are some limitations of this analysis: included studies were limited to Chinese and English language publications, and unpublished studies were not included; most of the included studies were MCC, and the quality of evidence for this design is not high; TaTME is a new surgical approach and physician's skill level is variable, which may have a potential impact on results.

## 5 Conclusion

The results of this meta-analysis show that TaTME was associated with a reduction in the positive CRM rate, TaTME thus could achieve complete tumor resection and improve long-term survival of patients with mid- and low-rectal cancer.

## Author contributions

Conception and design of the study: Tiankang Guo and Xiongfei Yang. Studies selection: Penghui Jin, Lidong Hu, and Dongping Hu. Data extraction: Penghui Jin and Wenhan Liu. Statistical analyses: Penghui Jin and Weisheng Zhang. Wrote the paper: Penghui Jin. The paper was revised and approved by Dongping Hu.

**Data curation:** Dongping Hu, Penghui Jin, Lidong Hu, Wenhan Liu.

**Formal analysis:** Weisheng Zhang.

**Investigation:** Lidong Hu.

**Methodology:** Dongping Hu, Penghui Jin.

**Resources:** Weisheng Zhang.

**Software:** Dongping Hu, Penghui Jin.

**Supervision:** Xiongfei Yang, Tiankang guo.

**Writing – original draft:** Dongping Hu, Penghui Jin.

**Writing – review and editing:** tiankang guo.
